# Supplementary material for: Convergent changes in muscle metabolism depend on duration of high-altitude ancestry across Andean waterfowl
Source: eLife. 2020 Jul 30;9:e56259. doi: 10.7554/eLife.56259 (PMC7494360; doi:10.7554/eLife.56259)
Supplement: Supplementary file 1. — (a) Maximal activities (µmol/g tissue/min), body mass (g) and myoglobin (Mb; mg/g tissue) concentration in pectoralis muscle. (b) Two-factor ANOVA results of maximal activities (µmol/g tissue/min), mass (g) and myoglobin (Mb; mg/g tissue) concentration in pectoralis muscle. (c) Two-factor ANOVA results of maximal activities (µmol/g tissue/min), mass (g) and myoglobin (Mb; mg/g tissue) concentration in pectoralis muscle excluding data for ruddy ducks from the subfamily Oxyurinae. (d) Test of covariance for enzyme activity (µmol/g tissue/min) or myoglobin content (Mb; mg/g tissue) and body mass (g). (e) Test of covariance for enzyme activity (µmol/g tissue/min) or myoglobin content (Mb; mg/g tissue) and body mass (g) excluding data for ruddy ducks from the subfamily Oxyurinae. (f) Correlation analyses of phylogenetic independent contrasts of bird mass (g), myoglobin (Mb) content (mg/g tissue), or enzyme activity (µmol/g tissue/min) versus altitude (m). (g) Correlation analyses of phylogenetic independent contrasts of bird mass (g), myoglobin (Mb) content (mg/g tissue), or enzyme activity (µmol/g tissue/min) versus altitude (m) excluding data for ruddy ducks from the subfamily Oxyurinae. (h) Assay conditions for enzymatic measurements. (i) List of GenBank gene accession numbers for mtDNA control region used in the construction of the phylogenetic tree. (j) Maximal activities (µmol/g tissue/min) in pectoralis muscle from surface, intermediate and deep tissue sampling locations. [file elife-56259-supp1.docx]

**Supplementary File 1a**.Maximal activities (µmol/g tissue/min), body mass (g) and myoglobin (Mb; mg/g tissue) concentration in pectoralis muscle.

|  | **Cinnamon teal** | | | **Yellow-billed**  **pintail** | | | **Ruddy duck** | | | **Crested duck** | | | | | **Puna teal (H)**  **Silver teal (L)** | | | | | **Speckled teal** | | | | **Andean goose (H)**  **Magellan goose (L)** | | | | | |
| --- | --- | --- | --- | --- | --- | --- | --- | --- | --- | --- | --- | --- | --- | --- | --- | --- | --- | --- | --- | --- | --- | --- | --- | --- | --- | --- | --- | --- | --- |
|  | **LA** | **HA** | **LA** | | **HA** | **LA** | | **HA** | **LA** | | | **HA** | | **LA** | | | **HA** | | **LA** | | | **HA** | | | **LA** | | **HA** | |  |
| Mass | 515.5  ± 24.5 | 510.83  ± 9.57 | 793.0  ± 18.46 | | 685.83  ± 15.1 | 631.5  ± 18.32 | | 824.17  ± 38.91 | 954.5  ± 29.8 | | | 1038.63  ± 39.35 | | 435.0  ± 9.43 | | | 468.57  ± 9.31 | | 435.5  ± 12.17 | | | 413.75  ± 12.23 | | | 2701.7  ± 221.36 | | 2503.85  ± 150.28 | |  |
| Mb | 4.80  ± 0.45 | 5.40  ± 0.36 | 9.96  ± 0.23 | | 9.41  ± 0.45 | 7.24  ± 0.35 | | 6.88  ± 0.44 | 7.37  ± 0.46 | | | ****9.70***  ***± 0.67*** | | 6.01  ± 0.24 | | | 7.29  ± 0.49 | | 6.58  ± 0.55 | | | 7.99  ± 0.47 | | | 8.77  ± 0.80 | | 9.66  ± 0.82 | |  |
| ***Carbohydrate metabolism*** | | | | | | | | | | | | | | | | | | | | | | | | | | | | |  |
| HK | 0.399  ± 0.050 | ****0.881***  ***± 0.038*** | 0.398  ± 0.068 | | ****0.897***  ***± 0.093*** | 1.281  ± 0.156 | | 1.184  ± 0.114 | 0.474  ± 0.078 | | | ****1.686***  ***± 0.148*** | | 0.410  ± 0.046 | | | 0.565  ± 0.110 | | 0.359  ± 0.057 | | | 0.439  ± 0.057 | | | 0.264  ± 0.089 | | 0.512  ± 0.063 | |  |
| PK | 515.69  ± 43.09 | 464.12  ± 32.28 | 566.23  ± 42.57 | | 504.04  ± 22.48 | 327.60  ± 13.43 | | 398.60  ± 26.40 | 581.74  ± 55.29 | | | 449.22  ± 39.51 | | 574.09  ± 36.16 | | | 502.06  ± 23.70 | | 546.62 ± 46.95 | | | ****280.51***  ***± 28.31*** | | | 372.27  ± 32.40 | | 393.56  ± 20.52 | |  |
| LDH | 348.51  ± 17.22 | 307.71  ± 12.20 | 359.57  ± 16.94 | | 317.58  ± 14.28 | 407.24  ± 34.14 | | ****227.49***  ***± 12.44*** | 363.77  ± 25.26 | | | ****249.27***  ***± 11.12*** | | 425.06  ± 23.63 | | | ****332.94***  ***± 13.56*** | | 392.79 ± 28.31 | | | ****290.54***  ***± 11.56*** | | | 379.27  ± 19.91 | | ****279.66***  ***± 7.98*** | |  |
| ***Citric acid cycle*** | | | | | | | | | | |  | |  | | |  | |  | | |  | |  | | |  | |  |  |
| CS | 76.23  ± 7.42 | 94.91  ± 6.87 | 76.01  ± 7.04 | | 83.17  ± 5.41 | 115.40  ± 7.82 | | 102.97  ± 7.95 | 83.79  ± 6.70 | | | 95.80  ± 5.50 | | 81.58  ± 10.88 | | | 65.09  ± 5.24 | | 87.02  ± 10.88 | | | 95.82  ± 6.84 | | | 76.87  ± 4.87 | | 74.33  ± 5.29 | |  |
| IDH | 19.52  ± 1.50 | 23.06  ± 1.17 | 22.81  ± 0.53 | | 21.56  ± 1.30 | 22.65  ± 2.22 | | 19.53  ± 1.37 | 15.67  ± 1.53 | | | 19.07  ± 2.14 | | 20.45  ± 1.18 | | | 17.12  ± 1.38 | | 23.81  ± 0.88 | | | 20.75  ± 1.42 | | | 26.41  ± 3.30 | | 24.10  ± 1.70 | |  |
| MDH | 749.35  ± 42.13 | 873.41  ± 53.07 | 804.34  ± 84.57 | | 696.72  ± 84.34 | 665.72  ± 37.88 | | 699.00  ± 51.59 | 809.19  ± 66.10 | | | 801.91  ± 46.46 | | 819.14  ± 62.41 | | | 634.06  ± 90.62 | | 897.16  ± 81.00 | | | 699.31  ± 68.05 | | | 957.75  ± 84.89 | | 782.28  ± 50.54 | |  |
| ***Electron transport chain*** | | | | | | | | | | |  | |  | | |  | |  | | |  | |  | | |  | |  |  |
| CI | 5.77  ± 0.87 | 5.66  ± 1.14 | 1.74  ± 0.40 | | 1.26  ± 0.22 | 1.30  ± 0.19 | | 2.21  ± 0.43 | 1.86  ± 0.41 | | | 3.48  ± 0.56 | | 3.71  ± 0.66 | | | 5.25  ± 0.82 | | 1.56  ± 0.39 | | | 2.02  ± 0.27 | | | 2.67  ± 0.85 | | 2.63  ± 0.29 | |  |
| CII | 3.41  ± 0.27 | 4.5  ± 0.28 | 4.85  ± 0.39 | | 4.78  ± 0.19 | 5.34  ± 0.18 | | 4.23  ± 0.18 | 4.40  ± 0.27 | | | 5.10  ± 0.28 | | 3.66  ± 0.29 | | | 3.84  ± 0.27 | | 3.38  ± 0.29 | | | ****5.08***  ***± 0.29*** | | | 3.61  ± 0.19 | | 4.00  ± 0.23 | |  |
| CIV | 39.88  ± 6.67 | ****25.69***  ***± 2.44*** | 61.36  ± 3.61 | | ****24.42***  ***± 3.12*** | 10.77  ± 1.31 | | ****26.75***  ***± 4.04*** | 58.41  ± 2.14 | | | ****26.64***  ***± 2.92*** | | 55.92  ± 2.72 | | | ****24.79***  ***± 3.66*** | | 56.86  ± 1.97 | | | ****25.99***  ***± 3.36*** | | | 72.63  ± 5.64 | | ****21.62***  ***± 2.86*** | |  |
| ATPsyn | 7.74  ± 1.65 | 9.59  ± 2.36 | 7.63  ± 2.31 | | 11.09  ± 3.37 | 10.03  ± 2.06 | | 7.20  ± 1.78 | 6.93  ± 1.34 | | | 14.01  ± 4.55 | | 7.86  ± 1.33 | | | ****15.73***  ***± 1.90*** | | 6.66  ± 1.49 | | | ****18.58***  ***± 3.08*** | | | 6.10  ± 2.50 | | ****18.84***  ***± 2.54*** | |  |
| ***Fatty acid metabolism*** | | | | | | | | | | |  | |  | | |  | |  | | |  | |  | | |  | |  |  |
| HOAD | 15.23  ± 1.03 | ****22.74***  ***± 1.11*** | 11.53  ± 0.73 | | ****25.37***  ***± 1.11*** | 52.87  ± 0.94 | | ****29.39***  ***± 1.48*** | 8.50  ± 0.44 | | | ****16.31***  ***± 1.15*** | | 11.74  ± 0.52 | | | ****19.21***  ***± 0.81*** | | 11.74  ± 0.74 | | | ****16.46***  ***± 1.17*** | | | 11.29  ± 0.53 | | ****13.73***  ***± 0.49*** | |  |
| ***Adenylate metabolism*** | | | | | | | | | | |  | |  | | |  | |  | | |  | |  | | |  | |  |  |
| AK | 220.53  ± 8.10 | 183.06  ± 7.52 | 245.65  ± 17.16 | | 268.57  ± 9.68 | 180.53  ± 11.90 | | 175.45  ± 9.49 | 267.72  ± 11.58 | | | 241.08  ± 7.62 | | 269.10  ± 12.70 | | | 265.93  ± 17.09 | | 251.08  ± 21.29 | | | 234.40  ± 6.29 | | | 296.86  ± 47.21 | | 231.79  ± 7.34 | |  |
| CK | 65.68  ± 4.49 | ****36.05***  ***± 2.17*** | 71.42  ± 5.78 | | ****42.74***  ***± 5.12*** | 52.38  ± 6.37 | | ****31.32***  ***± 5.48*** | 67.01  ± 4.13 | | | ****27.81***  ***± 5.39*** | | 63.86  ± 7.95 | | | ****32.45***  ***± 4.87*** | | 78.08  ± 7.50 | | | ****41.90***  ***± 6.23*** | | | 26.79  ± 1.17 | | ****13.84***  ***± 2.16*** | |  |

Values are given in as the mean ± SEM (*n* = 8-12). * - Significant pairwise differences between the high-altitude and low-altitude populations within a high-low pair in Bonferroni post-tests (P < 0.05). List of abbreviations: HA = high altitude; LA = low altitude; Mb = myoglobin; HK = hexokinase; PK = pyruvate kinase; LDH = lactate dehydrogenase; CS = citrate synthase; IDH = isocitrate dehydrogenase; MDH = malate dehydrogenase; CI = complex 1 (syn. NADH:ubiquinone oxidoreductase); CII = complex 2 (syn. succinate dehydrogenase); CIV = complex IV (syn. cytochrome c oxidase); ATPsyn = F_O_F_1_ ATP synthase; HOAD = 3-hydroxyacyl-CoA dehydrogenase; AK = adenylate kinase; and CK = creatine kinase.

**Supplementary File 1b**.Two-factor ANOVA results of maximal activities (µmol/g tissue/min), mass (g) and myoglobin (Mb; mg/g tissue) concentration in pectoralis muscle.

|  | **Altitude** | **Species** | **Interaction** |
| --- | --- | --- | --- |
| Mass | F_1,120_ = 0.00135, P = 0.9707 | F_6,120_ = 50.35, **P < 0.0001** | F_6,120_ = 0.3430, P = 0.9127 |
| Mb | F_1,120_ = 7.358, **P = 0.0077** | F_6,120_ = 16.82, **P < 0.0001** | F_6,120_ = 1.686, P = 0.1301 |
| ***Carbohydrate metabolism*** | | | |
| HK | F_1,120_ = 49.80, **P < 0.0001** | F_6,120_ = 23.31, **P < 0.0001** | F_6,120_ = 9.633, **P < 0.0001** |
| PK | F_1,120_ = 13.57, **P = 0.0003** | F_6,120_ = 8.669, **P < 0.0001** | F_6,120_ = 4.641, **P = 0.0003** |
| LDH | F_1,120_ = 82.29, **P < 0.0001** | F_6,120_ = 2.721, **P = 0.0163** | F_6,120_ = 2.854, **P = 0.0124** |
| ***Citric acid cycle*** | | | |
| CS | F_1,120_ = 0.310, P = 0.5787 | F_6,120_ = 5.583, **P < 0.0001** | F_6,120_ = 1.602, P = 0.1525 |
| IDH | F_1,120_ = 0.916, P = 0.3406 | F_6,120_ = 4.436, **P = 0.0004** | F_6,120_ = 1.583, P = 0.1577 |
| MDH | F_1,120_ = 3.993, **P = 0.0480** | F_6,120_ = 1.630, P = 0.1445 | F_6,120_ = 1.650, P = 0.1393 |
| ***Electron transport chain*** | | | |
| CI | F_1,120_ = 3.137, P = 0.0791 | F_6,120_ = 14.46, **P < 0.0001** | F_6,120_ = 0.9882, P = 0.4365 |
| CII | F_1,120_ = 7.836, **P = 0.0060** | F_6,120_ = 5.808, **P < 0.0001** | F_6,120_ = 5.190, **P < 0.0001** |
| CIV | F_1,120_ = 192.6, **P < 0.0001** | F_6,120_ = 15.06, **P < 0.0001** | F_6,120_ = 18.95, **P < 0.0001** |
| ATPsyn | F_1,120_ = 19.45, **P < 0.0001** | F_6,120_ = 0.780, P = 0.5874 | F_6,120_ = 2.362, **P = 0.0342** |
| ***Fatty acid metabolism*** | | | |
| HOAD | F_1,120_ = 31.93, **P < 0.0001** | F_6,120_ = 221.1, **P < 0.0001** | F_6,120_ = 79.95, **P < 0.0001** |
| ***Adenylate metabolism*** | | | |
| AK | F_1,120_ = 3.910, P = 0.0503 | F_6,120_ = 9.216, **P < 0.0001** | F_6,120_ = 1.207, P = 0.3072 |
| CK | F_1,120_ = 91.95, **P < 0.0001** | F_6,120_ = 11.08, **P < 0.0001** | F_6,120_ = 1.301, P = 0.2620 |

Two‐factor ANOVA was used to evaluate the main effects and interactions of altitude on enzyme activity (high- vs. low-altitude populations across all species) and species on enzyme activity (species-specific differences across all altitudes). List of abbreviations: HA = high altitude; LA = low altitude; Mb = myoglobin; HK = hexokinase; PK = pyruvate kinase; LDH = lactate dehydrogenase; CS = citrate synthase; IDH = isocitrate dehydrogenase; MDH = malate dehydrogenase; CI = complex 1 (syn. NADH:ubiquinone oxidoreductase); CII = complex 2 (syn. succinate dehydrogenase); CIV = complex IV (syn. cytochrome c oxidase); ATPsyn = F_O_F_1_ ATP synthase; HOAD = 3-hydroxyacyl-CoA dehydrogenase; AK = adenylate kinase; and CK = creatine kinase.

**Supplementary File 1c**.Two-factor ANOVA results of maximal activities (µmol/g tissue/min), mass (g) and myoglobin (Mb; mg/g tissue) concentration in pectoralis muscle excluding data for ruddy ducks from the subfamily *Oxyurinae*.

|  | **Altitude** | **Species** | **Interaction** |
| --- | --- | --- | --- |
| Mass | F_1,106_ = 0.1498, P = 0.6995 | F_5,106_ = 54.98, **P < 0.0001** | F_5,106_ = 0.2035, P = 0.9604 |
| Mb | F_1,106_ = 9.404, **P = 0.0027** | F_5,106_ = 19.00, **P < 0.0001** | F_5,106_ = 1.458, P = 0.2098 |
| ***Carbohydrate metabolism*** | | | |
| HK | F_1,106_ = 76.19, **P < 0.0001** | F_5,106_ = 16.92, **P < 0.0001** | F_5,106_ = 10.84, **P < 0.0001** |
| PK | F_1,106_ = 19.56, **P < 0.0001** | F_5,106_ = 6.397, **P < 0.0001** | F_5,106_ = 3.531, **P = 0.0054** |
| LDH | F_1,106_ = 63.27, **P < 0.0001** | F_5,106_ = 3.590, **P = 0.0049** | F_5,106_ = 1.629, P = 0.1587 |
| ***Citric acid cycle*** | | | |
| CS | F_1,106_ = 1.256, P = 0.2649 | F_5,106_ = 2.232, P = 0.0564 | F_5,106_ = 1.528, P = 0.1872 |
| IDH | F_1,106_ = 0.2803, P = 0.5976 | F_5,106_ = 5.779, **P < 0.0001** | F_5,106_ = 1.847, P = 0.1100 |
| MDH | F_1,106_ = 5.050, **P = 0.0267** | F_5,106_ = 1.018, P = 0.4111 | F_5,106_ = 1.626, P = 0.1594 |
| ***Electron transport chain*** | | | |
| CI | F_1,106_ = 2.023, P = 0.1579 | F_5,106_ = 14.55, **P < 0.0001** | F_5,106_ = 1.076, P = 0.3781 |
| CII | F_1,106_ = 16.72, **P = 0.0002** | F_5,106_ = 5.355, **P < 0.0001** | F_5,106_ = 2.640, **P = 0.0273** |
| CIV | F_1,106_ = 257.2, **P < 0.0001** | F_5,106_ = 3.583, **P = 0.0049** | F_5,106_ = 5.649, **P = 0.0001** |
| ATPsyn | F_1,106_ = 25.13, **P < 0.0001** | F_5,106_ = 0.6869, P = 0.6344 | F_5,106_ = 1.439, P = 0.2164 |
| ***Fatty acid metabolism*** | | | |
| HOAD | F_1,106_ = 185.6, **P < 0.0001** | F_5,106_ = 19.11, **P < 0.0001** | F_5,106_ = 8.531, **P < 0.0001** |
| ***Adenylate metabolism*** | | | |
| AK | F_1,106_ = 4.058, **P = 0.0465** | F_5,106_ = 4.506, **P = 0.0009** | F_5,106_ = 1.341, P = 0.2527 |
| CK | F_1,106_ = 90.95, **P < 0.0001** | F_5,106_ = 13.75, **P < 0.0001** | F_5,106_ = 1.436, P = 0.2174 |

Two‐factor ANOVA was used to evaluate the main effects and interactions of altitude on enzyme activity (high- vs. low-altitude populations across all species) and species on enzyme activity (species-specific differences across all altitudes). List of abbreviations: HA = high altitude; LA = low altitude; Mb = myoglobin; HK = hexokinase; PK = pyruvate kinase; LDH = lactate dehydrogenase; CS = citrate synthase; IDH = isocitrate dehydrogenase; MDH = malate dehydrogenase; CI = complex 1 (syn. NADH:ubiquinone oxidoreductase); CII = complex 2 (syn. succinate dehydrogenase); CIV = complex IV (syn. cytochrome c oxidase); ATPsyn = F_O_F_1_ ATP synthase; HOAD = 3-hydroxyacyl-CoA dehydrogenase; AK = adenylate kinase; and CK = creatine kinase.

**Supplementary File 1d**.Test of covariance for enzyme activity (µmol/g tissue/min) or myoglobin content (Mb; mg/g tissue) and body mass (g).

|  | **Statistical results** | **Regression analysis** |  | |
| --- | --- | --- | --- | --- |
| Mb | F_1,12_ = 4.371, P = 0.0585 | r^2^ = 0.2670 |  | |
| ***Carbohydrate metabolism*** | | | | |
| HK | F_1,12_ = 0.2995, P = 0.5943 | r^2^ = 0.02435 |  | |
| PK | F_1,12_ = 1.301, P = 0.2762 | r^2^ = 0.09784 |  | |
| LDH | F_1,12_ = 0.1856, P = 0.6742 | r^2^ = 0.01523 |  | |
| ***Citric acid cycle*** | | | |  |
| CS | F_1,12_ = 0.9914, P = 0.3391 | r^2^ = 0.07631 |  | |
| IDH | F_1,12_ = 4.045, P = 0.0673 | r^2^ = 0.2521 |  | |
| MDH | F_1,12_ = 2.739, P = 0.1858 | r^2^ = 0.1858 |  | |
| ***Electron transport chain*** | | | | |
| CI | F_1,12_ = 0.2976, P = 0.5954 | r^2^ = 0.02420 |  | |
| CII | F_1,12_ = 0.4830, P = 0.5003 | r^2^ = 0.03870 |  | |
| CIV | F_1,12_ = 0.6840, P = 0.4244 | r^2^ = 0.05393 |  | |
| ATPsyn | F_1,12_ = 0.1366, P = 0.7181 | r^2^ = 0.01126 |  | |
| ***Fatty acid metabolism*** | | | | |
| HOAD | F_1,12_ = 0.7024, P = 0.4184 | r^2^ = 0.05529 |  | |
| ***Adenylate metabolism*** | | | | |
| AK | F_1,12_ = 1.217, P = 0.2916 | r^2^ = 0.09207 |  | |
| CK | F_1,12_ = 4.131, P = 0.0649 | r^2^ = 0.2561 |  | |

List of abbreviations: HA = high altitude; LA = low altitude; Mb = myoglobin; HK = hexokinase; PK = pyruvate kinase; LDH = lactate dehydrogenase; CS = citrate synthase; IDH = isocitrate dehydrogenase; MDH = malate dehydrogenase; CI = complex 1 (syn. NADH:ubiquinone oxidoreductase); CII = complex 2 (syn. succinate dehydrogenase); CIV = complex IV (syn. cytochrome c oxidase); ATPsyn = F_O_F_1_ ATP synthase; HOAD = 3-hydroxyacyl-CoA dehydrogenase; AK = adenylate kinase; and CK = creatine kinase.

**Supplementary File 1e**. Test of covariance for enzyme activity (µmol/g tissue/min) or myoglobin content (Mb; mg/g tissue) and body mass (g) excluding data for ruddy ducks from the subfamily *Oxyurinae*.

|  | **Statistical Results** | **Regression Analysis** |  | |
| --- | --- | --- | --- | --- |
| Mb | F_1,10_ = 3.558, P = 0.0886 | r^2^ = 0.2624 |  | |
| ***Carbohydrate metabolism*** | | | | |
| HK | F_1,10_ = 0.1284, P = 0.7275 | r^2^ = 0.01268 |  | |
| PK | F_1,10_ = 2.141, P = 0.1741 | r^2^ = 0.1763 |  | |
| LDH | F_1,10_ = 0.1868, P = 0.6742 | r^2^ = 0.01834 |  | |
| ***Citric acid cycle*** | | | |  |
| CS | F_1,10_ = 3.851, P = 0.3730 | r^2^ = 0.08000 |  | |
| IDH | F_1,10_ = 4.045, P = 0.0781 | r^2^ = 0.2780 |  | |
| MDH | F_1,10_ = 2.210, P = 0.1680 | r^2^ = 0.1810 |  | |
| ***Electron transport chain*** | | | | |
| CI | F_1,10_ = 0.4597, P = 0.5132 | r^2^ = 0.04395 |  | |
| CII | F_1,10_ = 0.2776, P = 0.6098 | r^2^ = 0.02701 |  | |
| CIV | F_1,10_ = 0.4159, P = 0.5335 | r^2^ = 0.03993 |  | |
| ATPsyn | F_1,10_ = 0.09083, P = 0.7693 | r^2^ = 0.009002 |  | |
| ***Fatty acid metabolism*** | | | | |
| HOAD | F_1,10_ = 1.036, P = 0.3327 | r^2^ = 0.09388 |  | |
| ***Adenylate metabolism*** | | | | |
| AK | F_1,10_ = 1.113, P = 0.3162 | r^2^ = 0.1002 |  | |
| CK | F_1,10_ = 3.851, P = 0.0781 | r^2^ = 0.2780 |  | |

List of abbreviations: HA = high altitude; LA = low altitude; Mb = myoglobin; HK = hexokinase; PK = pyruvate kinase; LDH = lactate dehydrogenase; CS = citrate synthase; IDH = isocitrate dehydrogenase; MDH = malate dehydrogenase; CI = complex 1 (syn. NADH:ubiquinone oxidoreductase); CII = complex 2 (syn. succinate dehydrogenase); CIV = complex IV (syn. cytochrome c oxidase); ATPsyn = F_O_F_1_ ATP synthase; HOAD = 3-hydroxyacyl-CoA dehydrogenase; AK = adenylate kinase; and CK = creatine kinase.

**Supplementary File 1f**. Correlation analyses of phylogenetic independent contrasts of bird mass (g), myoglobin (Mb) content (mg/g tissue), or enzyme activity (µmol/g tissue/min) *versus* altitude (m).

|  | **Pearson product-moment correlation coefficient** | **F_2,10_, P** | **R^2^** |
| --- | --- | --- | --- |
| Mass | -0.0683 | F_2,12_ = 0.0562, P = 0.8166 | r^2^ = 0. 0047 |
| Mb | 0.4943 | F_2,12_ = 3.8803, P = 0.0724 | r^2^ = 0.2443 |
| ***Carbohydrate metabolism*** | | | |
| HK | 0. 7160 | F_2,12_ = 12.6211, **P = 0.0040** | r^2^ = 0. 5126 |
| PK | -0.7455 | F_2,12_ = 15.0169, **P = 0.0021** | r^2^ = 0. 5558 |
| LDH | -0.8655 | F_2,12_ = 35.8287, **P < 0.0001** | r^2^ = 0.7491 |
| ***Citric acid cycle*** | | | |
| CS | 0.6128 | F_2,12_ = 7.2177, **P = 0.0198** | r^2^ = 0.3756 |
| IDH | -0.2884 | F_2,12_ = 1. 0886, P = 0.3173 | r^2^ = 0.0832 |
| MDH | -0.5350 | F_2,12_ = 4.8124, **P = 0.0487** | r^2^ = 0.2862 |
| ***Electron transport chain*** | | | |
| CI | 0.3024 | F_2,12_ = 1.2082, P = 0.2933 | r^2^ = 0.0915 |
| CII | 0.6070 | F_2,12_ = 7.0016, **P = 0.0213** | r^2^ = 0.3685 |
| CIV | -0.8502 | F_2,12_ = 31.2905, **P = 0.0001** | r^2^ = 0.7228 |
| ATPsyn | 0.7842 | F_2,12_ = 19.1688, **P = 0.0009** | r^2^ = 0.6150 |
| ***Fatty acid metabolism*** | | | |
| HOAD | 0.4356 | F_2,12_ = 2.8100, P = 0.1195 | r^2^ = 0.1897 |
| ***Adenylate metabolism*** | | | |
| AK | -0.3552 | F_2,12_ = 1.7324, P = 0.2127 | r^2^ = 0.1262 |
| CK | -0.9777 | F_2,12_ = 259.6513, **P < 0.0001** | r^2^ = 0.9558 |

List of abbreviations: HA = high altitude; LA = low altitude; Mb = myoglobin; HK = hexokinase; PK = pyruvate kinase; LDH = lactate dehydrogenase; CS = citrate synthase; IDH = isocitrate dehydrogenase; MDH = malate dehydrogenase; CI = complex 1 (syn. NADH:ubiquinone oxidoreductase); CII = complex 2 (syn. succinate dehydrogenase); CIV = complex IV (syn. cytochrome c oxidase); ATPsyn = F_O_F_1_ ATP synthase; HOAD = 3-hydroxyacyl-CoA dehydrogenase; AK = adenylate kinase; and CK = creatine kinase.

**Supplementary File 1g**. Correlation analyses of phylogenetic independent contrasts of bird mass (g), myoglobin (Mb) content (mg/g tissue), or enzyme activity (µmol/g tissue/min) *versus* altitude (m) excluding data for ruddy ducks from the subfamily *Oxyurinae*.

|  | **Pearson product-moment correlation coefficient** | **F_2,10_, P** | **R^2^** |
| --- | --- | --- | --- |
| Mass | -0.1735 | F_2,12_ = 0.3103, P = 0.5898 | r^2^ = 0.0301 |
| Mb | 0.5527 | F_2,12_ = 4.3990, P = 0.0624 | r^2^ = 0.3055 |
| ***Carbohydrate metabolism*** | | | |
| HK | 0.7735 | F_2,12_ = 14.90, **P = 0.0032** | r^2^ = 0.5983 |
| PK | -0.8340 | F_2,12_ = 22.83904, **P = 0.0007** | r^2^ = 0.6955 |
| LDH | -0.9345 | F_2,12_ = 68.9180, **P < 0.0001** | r^2^ = 0.8733 |
| ***Citric acid cycle*** | | | |
| CS | 0.4035 | F_2,12_ = 3.2194, P = 0.0916 | r^2^ = 0.1615 |
| IDH | -0.2070 | F_2,12_ = 0.4476, P = 0.5186 | r^2^ = 0.0428 |
| MDH | -0.5855 | F_2,12_ = 5.2158, **P = 0.0455** | r^2^ = 0.3428 |
| ***Electron transport chain*** | | | |
| CI | 0.2198 | F_2,12_ = 0.5076, P = 0.4925 | r^2^ = 0.0483 |
| CII | 0.7767 | F_2,12_ = 15.2029, **P = 0.0030** | r^2^ = 0.6032 |
| CIV | -0.9595 | F_2,12_ = 116.0305, **P < 0.0001** | r^2^ = 0.9207 |
| ATPsyn | 0.8653 | F_2,12_ = 29.8020, **P = 0.0003** | r^2^ = 0.7488 |
| ***Fatty acid metabolism*** | | | |
| HOAD | 0.8837 | F_2,12_ = 35.6387, **P = 0.0001** | r^2^ = 0.7809 |
| ***Adenylate metabolism*** | | | |
| AK | -0.3606 | F_2,12_ = 1.4946, P = 0.2495 | r^2^ = 0.1300 |
| CK | -0.9770 | F_2,12_ = 210.1855, **P < 0.0001** | r^2^ = 0.9546 |

List of abbreviations: HA = high altitude; LA = low altitude; Mb = myoglobin; HK = hexokinase; PK = pyruvate kinase; LDH = lactate dehydrogenase; CS = citrate synthase; IDH = isocitrate dehydrogenase; MDH = malate dehydrogenase; CI = complex 1 (syn. NADH:ubiquinone oxidoreductase); CII = complex 2 (syn. succinate dehydrogenase); CIV = complex IV (syn. cytochrome c oxidase); ATPsyn = F_O_F_1_ ATP synthase; HOAD = 3-hydroxyacyl-CoA dehydrogenase; AK = adenylate kinase; and CK = creatine kinase.

**Supplementary File 1h.** Assay conditions for enzymatic measurements.

|  | **λ (nm)** | **ε (mmol^-1^ cm^-1^)** | | **pH** | **Substrates** | **Other reagents** | **Specific inhibitor** | | **Coupling enzymes** |
| --- | --- | --- | --- | --- | --- | --- | --- | --- | --- |
| ***Carbohydrate metabolism*** | | |  | | |  | |  |  |
| **HK** | 340 | 6.22 | | 7.2 | ***Glucose (10 mM)**  Mg·ATP (3 mM)  MgCl_2_ (10 mM)  NADP^+^ (1.5 mM) | --- | --- | | 1 U G6PDH |
| **PK** | 340 | 6.22 | | 7.2 | ***PEP (10 mM)**  Mg·ADP (2.5 mM) | --- | --- | | 1 U LDH |
| **LDH** | 340 | 6.22 | | 7.2 | ***Pyruvate (5 mM)**  NADH (0.15 mM) | --- | --- | | --- |
| ***Citric acid cycle*** | | |  | | |  | |  |  |
| **CS** | 412 | 14.15 | | 8.0 | ***Oxaloacetate (0.5 mM)**  Acetyl CoA (0.15 mM)  DTNB (0.15 mM) | --- | --- | | --- |
| **IDH** | 340 | 6.22 | | 8.0 | ***Isocitrate (5 mM)**  NADP^+^ (1.5 mM) | --- | --- | | --- |
| **MDH** | 340 | 6.22 | | 8.0 | ***Oxaloacetate (0.5 mM)**  NADP^+^ (1.5 mM) | --- | --- | | --- |
| ***Electron transport chain*** | | |  | | |  | |  |  |
| **CI** | 340 | 6.22 | | 7.5 | CoQ_10_ (0.06 mM)  NADH (0.15 mM) | BSA (3mg ml^-1^)  KCN (0.3 mM) | ***Rotenone (0.02 mM)** | | --- |
| **CII** | 600 | 21.9 | | 7.5 | ***Succinate (20 mM)**  DCPIP (XX mM)  DUB (XX mM) | KCN (0.3 mM) | --- | | --- |
| **CIV** | 550 | 28.5 | | 7.0 | **†CytcH_2_ (2 mM)** | --- | --- | | --- |
| **ATPsyn** | 340 | 6.22 | | 7.5 | Mg·ADP (2.5 mM)  MgCl_2_ (10 mM)  PEP (10 mM)  Glucose (10 mM)  NADP^+^ (1.5 mM) | --- | ***Oligomycin (0.01 mM)** | | 1 U HK  1 U G6PDH |
| ***Fatty acid metabolism*** | | |  | | |  | |  |  |
| **HOAD** | 340 | 6.22 | | 7.2 | ***Acetoacetyl CoA (0.15 mM)**  NADH (0.15 mM) | --- | --- | | --- |
| ***Adenylate metabolism*** | | |  | | |  | |  |  |
| **AK** | 340 | 6.22 | | 7.2 | ***Mg·ADP (2.5 mM)**  MgCl_2_ (10 mM)  PEP (10 mM)  Glucose (10 mM)  NADP^+^ (1.5 mM) | --- | --- | | 1 U HK  1 U G6PDH |
| **CK** | 340 | 6.22 | | 7.2 | ***Creatine (15 mM)**  Mg·ATP (6 mM)  MgCl_2_ (10 mM)  PEP (15 mM)  NADH (0.3 mM) | --- | --- | | 2 U PK  2 U LDH |

* - Substrate omitted in measurement of background activity. † - Substrate auto-oxidation measured as background activity. List of abbreviations: Mb = myoglobin; HK = hexokinase; PK = pyruvate kinase; LDH = lactate dehydrogenase; CS = citrate synthase; IDH = isocitrate dehydrogenase; MDH = malate dehydrogenase; CI = complex 1 (syn. NADH:ubiquinone oxidoreductase); CII = complex 2 (syn. succinate dehydrogenase); CIV = complex IV (syn. cytochrome c oxidase); ATPsyn = F_O_F_1_ ATP synthase; HOAD = 3-hydroxyacyl-CoA dehydrogenase; AK = adenylate kinase; CK = creatine kinase; G6PDH = glucose-6-phosphate dehydrogenase; ATP = adenosine triphosphate; ADP = adenosine diphosphate; NADP = nicotinamide adenine dinucleotide phosphate; NADH = reduced nicotinamide adenine dinucleotide; CoA = coenzyme A; DTNB = Ellman's reagent (syn. 5,5'-dithiobis-2-nitrobenzoic acid); CoQ10 = ubiquinone; BSA = bovine serum albumin; DCPIP = 2,6-dichlorophenolindophenol; DUB = decylubiquinone (syn. 2,3-dimethoxy-5-methyl-6-decyl-1,4-benzoquinone); CytcH2 = reduced cytochrome c; and PEP = phosphoenolpyruvate

**Supplementary File 1i**. List of GenBank gene accession numbers for mtDNA control region used in the construction of the phylogenetic tree.

| **Species** | **Accession Numbers** |
| --- | --- |
|  |  |
| Yellow-billed Pintail | FJ618397-FJ618512 |
| Cinnamon Teal | JF914653-JF914754 |
| Ruddy Duck | AY747742-AY747751; AY747756-AY747778; AM084943-AM084997; JX910949-JX910971 |
| Crested Duck | HM063481-HM063503; JN833791-JN833847 |
| Puna Teal/Silver Teal | MN734269-MN734345 |
| Speckled Teal | JN223305-JN22337; MG520106-MG520175 |
| Andean Goose/Magellan Goose | KC109071-KC109080 |
|  |  |

**Supplementary File 1j**. Maximal activities (µmol/g tissue/min) in pectoralis muscle from surface, intermediate and deep tissue sampling locations.

|  | **Cinnamon teal** | | | **Yellow-billed**  **pintail** | | | **Ruddy duck** | | | **Crested duck** | | | | | **Puna teal (H)**  **Silver teal (L)** | | | | | **Speckled teal** | | | | **Andean goose (H)**  **Magellan goose (L)** | | | | | |
| --- | --- | --- | --- | --- | --- | --- | --- | --- | --- | --- | --- | --- | --- | --- | --- | --- | --- | --- | --- | --- | --- | --- | --- | --- | --- | --- | --- | --- | --- |
|  | **LA** | **HA** | **LA** | | **HA** | **LA** | | **HA** | **LA** | | | **HA** | | **LA** | | | **HA** | | **LA** | | | **HA** | | | **LA** | | **HA** | |  |
| ***Carbohydrate metabolism*** | | | | | | | | | | | | | | | | | | | | | | | | | | | | |  |
| HK  (S) | 0.318  ± 0.045 | 0.901  ± 0.073 | 0.421  ± 0.074 | | 1.169  ± 0.057 | 1.661  ± 0.047 | | 1.507  ± 0.070 | 0.452  ± 0.065 | | | 2.089  ± 0.080 | | 0.451  ± 0.059 | | | 0.570  ± 0.101 | | 0.353  ± 0.056 | | | 0.430  ± 0.043 | | |  | | 0.698  ± 0.014 | |  |
| HK  (I) | 0.442  ± 0.114 | 0.903  ± 0.041 | 0.479  ± 0.079 | | 0.874  ± 0.083 | 1.399  ± 0.117 | | 1.291  ± 0.071 | 0.551  ± 0.127 | | | 1.655  ± 0.100 | | 0.393  ± 0.038 | | | 0.571  ± 0.192 | | 0.331  ± 0.050 | | | 0.401  ± 0.072 | | | 0.264  ± 0.089 | | 0.407  ± 0.040 | |  |
| HK  (D) | 0.493  ± 0.082 | 0.830  ± 0.093 | 0.281  ± 0.024 | | 0.558  ± 0.027 | 0.660  ± 0.096 | | 0.785  ± 0.055 | 0.434  ± 0.059 | | | 1.279  ± 0.130 | | 0.387  ± 0.039 | | | 0.555  ± 0.063 | | 0.391  ± 0.069 | | | 0.486  ± 0.053 | | |  | | 0.425  ± 0.067 | |  |
| PK  (S) | 558.95  ± 30.44 | 565.24  ± 14.92 | 689.47  ± 26.66 | | 554.60  ± 14.84 | 361.09  ± 12.61 | | 483.95  ± 17.08 | 682.16  ± 26.22 | | | 524.42  ± 15.29 | | 677.11  ± 28.03 | | | 560.95  ± 12.84 | | 671.93  ± 34.62 | | | 378.27  ± 13.89 | | |  | | 469.77  ± 14.41 | |  |
| PK  (I) | 501.94  ± 29.78 | 476.95± 15.34 | 540.43  ± 21.81 | | 510.48  ± 15.93 | 339.09  ± 10.18 | | 392.00  ± 16.79 | 530.08  ± 16.39 | | | 430.99  ± 13.13 | | 554.63  ± 25.41 | | | 510.48  ± 15.93 | | 546.03  ± 42.05 | | | 249.26  ± 16.97 | | | 372.27  ± 32.40 | | 363.81  ± 5.93 | |  |
| PK  (D) | 413.41  ± 49.61 | 330.25  ± 13.43 | 468.79  ± 42.84 | | 401.44  ± 7.24 | 290.48  ± 8.55 | | 317.97  ± 16.07 | 532.99  ± 17.83 | | | 398.53  ± 10.19 | | 490.52  ± 28.02 | | | 436.27  ± 20.39 | | 421.92  ± 25.74 | | | 207.94  ± 15.00 | | |  | | 347.10  ± 8.32 | |  |
| LDH  (S) | 396.64  ± 20.93 | 368.38  ± 14.08 | 452.01  ± 21.69 | | 379.74  ± 21.82 | 556.93  ± 13.48 | | 267.23  ± 22.23 | 466.62  ± 38.90 | | | 312.53  ± 20.35 | | 525.48  ± 31.50 | | | 407.43  ± 12.94 | | 519.64  ± 32.13 | | | 352.66  ± 16.23 | | |  | | 289.58  ± 14.26 | |  |
| LDH  (I) | 315.22  ± 27.17 | 308.51  ± 10.12 | 337.72  ± 17.13 | | 307.04  ± 9.69 | 310.48  ± 15.93 | | 194.31  ± 16.02 | 359.76  ± 35.71 | | | 253.49  ± 6.69 | | 435.46  ± 32.46 | | | 332.08  ± 16.11 | | 394.62  ± 32.91 | | | 281.21  ± 12.20 | | | 379.27  ± 19.91 | | 257.09  ± 11.76 | |  |
| LDH  (D) | 270.71  ± 17.38 | 237.22  ± 10.70 | 288.98  ± 21.31 | | 228.91  ± 11.05 | 164.63  ± 5.75 | | 211.47  ± 14.37 | 264.92  ± 33.41 | | | 187.07  ± 6.18 | | 314.23  ± 29.22 | | | 259.15  ± 10.86 | | 264.11  ± 44.74 | | | 232.94  ± 13.73 | | |  | | 292.31  ± 14.04 | |  |
| ***Citric acid cycle*** | | | | | | | | | | |  | |  | | |  | |  | | |  | |  | | |  | |  |  |
| CS  (S) | 62.86  ± 5.99 | 74.35  ± 8.34 | 75.77  ± 4.28 | | 69.41  ± 5.42 | 127.31  ± 4.79 | | 101.38  ± 9.02 | 70.82  ± 7.12 | | | 82.17  ± 4.06 | | 52.69  ± 2.42 | | | 58.61  ± 3.88 | | 75.29  ± 3.12 | | | 86.13  ± 4.68 | | |  | | 62.24  ± 4.12 | |  |
| CS  (I) | 84.29  ± 5.44 | 101.65  ± 3.67 | 71.24  ± 3.41 | | 90.90  ± 3.13 | 86.50  ± 3.71 | | 86.92  ± 9.15 | 86.06  ± 5.59 | | | 103.22  ± 3.10 | | 95.78  ± 5.03 | | | 73.43  ± 3.46 | | 107.74  ± 4.71 | | | 106.59  ± 5.50 | | | 76.87  ± 4.87 | | 71.40  ± 2.60 | |  |
| CS  (D) | 100.25  ± 10.49 | 108.80  ± 4.94 | 81.02  ± 11.15 | | 90.91  ± 7.68 | 132.38  ± 4.44 | | 116.03  ± 9.48 | 94.49  ± 5.55 | | | 102.01  ± 5.37 | | 96.27  ± 10.46 | | | 64.75  ± 6.50 | | 78.02  ± 16.67 | | | 94.49  ± 9.10 | | |  | | 88.34  ± 4.50 | |  |
| IDH  (S) | 16.69  ± 1.15 | 19.87  ± 1.09 | 21.50  ± 0.52 | | 18.33  ± 1.41 | 17.46  ± 1.13 | | 17.68  ± 1.54 | 10.98  ± 0.63 | | | 17.83  ± 2.61 | | 18.19  ± 0.83 | | | 14.26  ± 0.53 | | 22.45  ± 0.70 | | | 18.00  ± 0.84 | | |  | | 18.06  ± 0.84 | |  |
| IDH  (I) | 21.21  ± 1.44 | 24.39  ± 0.91 | 23.13  ± 0.48 | | 23.16  ± 0.81 | 23.69  ± 3.05 | | 18.49  ± 2.24 | 14.43  ± 0.41 | | | 18.72  ± 2.23 | | 21.37  ± 0.63 | | | 17.29  ± 0.58 | | 23.84  ± 0.49 | | | 23.22  ± 0.48 | | | 26.41  ± 3.30 | | 23.52  ± 0.43 | |  |
| IDH  (D) | 24.62  ± 0.81 | 24.81  ± 1.25 | 23.81  ± 0.28 | | 23.83  ± 1.52 | 26.80  ± 0.57 | | 22.13  ± 1.23 | 21.61  ± 0.65 | | | 20.57  ± 0.91 | | 21.80  ± 0.53 | | | 19.84  ± 0.73 | | 25.16  ± 0.31 | | | 20.56  ± 0.86 | | |  | | 29.73  ± 0.95 | |  |
| MDH  (S) | 701.76  ± 38.96 | 808.69  ± 37.43 | 547.07  ± 65.37 | | 574.24  ± 91.46 | 616.35  ± 34.66 | | 721.10  ± 50.97 | 709.97  ± 41.80 | | | 700.67  ± 43.76 | | 624.44  ± 34.40 | | | 510.24  ± 38.39 | | 851.73  ± 52.24 | | | 687.81  ± 69.42 | | |  | | 670.42  ± 37.92 | |  |
| MDH  (I) | 746.65  ± 23.29 | 961.01  ± 68.77 | 855.41  ± 37.00 | | 737.10  ± 74.08 | 650.81  ± 42.82 | | 612.51  ± 75.20 | 778.40  ± 73.79 | | | 842.57  ± 37.59 | | 867.99  ± 40.18 | | | 675.15  ± 78.05 | | 917.49  ± 81.63 | | | 720.53  ± 76.88 | | | 957.75  ± 84.89 | | 812.59  ± 46.07 | |  |
| MDH  (D) | 897.52  ± 72.13 | 822.21  ± 32.08 | 1010.54  ± 70.97 | | 828.35  ± 114.36 | 728.51  ± 32.63 | | 738.67  ± 69.86 | 939.22  ± 61.70 | | | 854.05  ± 36.05 | | 945.53  ± 61.05 | | | 724.28  ± 120.20 | | 922.25  ± 106.79 | | | 689.58  ± 51.69 | | |  | | 863.83  ± 37.24 | |  |
| ***Electron transport chain*** | | | | | | | | | | |  | |  | | |  | |  | | |  | |  | | |  | |  |  |
| CI  (S) | 5.65  ± 1.00 | 4.29  ± 1.08 | 1.31  ± 0.28 | | 1.00  ± 0.20 | 1.24  ± 0.18 | | 1.98  ± 0.40 | 1.49  ± 0.34 | | | 2.85  ± 0.74 | | 3.48  ± 0.77 | | | 4.49  ± 0.62 | | 1.69  ± 0.61 | | | 1.42  ± 0.27 | | |  | | 1.85  ± 0.24 | |  |
| CI  (I) | 5.86  ± 0.60 | 5.66  ± 1.51 | 2.22  ± 0.83 | | 1.37  ± 0.24 | 1.29  ± 0.17 | | 2.36  ± 1.11 | 2.12  ± 0.67 | | | 4.26  ± 0.51 | | 3.23  ± 0.72 | | | 5.39  ± 0.71 | | 1.51  ± 0.49 | | | 2.07  ± 0.22 | | | 2.67  ± 0.85 | | 2.58  ± 0.16 | |  |
| CI  (D) | 6.22  ± 0.99 | 6.86  ± 0.79 | 1.84  ± 0.10 | | 1.43  ± 0.36 | 1.34  ± 0.24 | | 2.35  ± 0.47 | 2.11  ± 0.52 | | | 3.22  ± 0.22 | | 4.61  ± 0.93 | | | 6.05  ± 1.18 | | 1.52  ± 0.28 | | | 2.39  ± 0.26 | | |  | | 3.46  ± 0.14 | |  |
| CII  (S) | 2.94  ± 0.23 | 3.64  ± 0.32 | 3.88  ± 0.31 | | 4.36  ± 0.14 | 5.16  ± 0.18 | | 4.00  ± 0.29 | 4.56  ± 0.20 | | | 4.08  ± 0.13 | | 2.84  ± 0.08 | | | 3.30  ± 0.13 | | 3.07  ± 0.22 | | | 4.56  ± 0.22 | | |  | | 3.53  ± 0.16 | |  |
| CII  (I) | 3.57  ± 0.23 | 5.07  ± 0.18 | 5.36  ± 0.41 | | 5.27  ± 0.13 | 5.70  ± 0.17 | | 4.29  ± 0.10 | 4.51  ± 0.28 | | | 5.36  ± 0.14 | | 3.82  ± 0.26 | | | 4.26  ± 0.20 | | 3.71  ± 0.34 | | | 5.44  ± 0.32 | | | 3.61  ± 0.19 | | 3.89  ± 0.14 | |  |
| CII  (D) | 4.49  ± 0.12 | 4.84  ± 0.13 | 5.32  ± 0.28 | | 4.45  ± 0.12 | 5.17  ± 0.14 | | 4.42  ± 0.21 | 4.14  ± 0.34 | | | 5.85  ± 0.10 | | 4.31  ± 0.26 | | | 4.04  ± 0.34 | | 3.35  ± 0.30 | | | 5.26  ± 0.22 | | |  | | 4.57  ± 0.22 | |  |
| CIV  (S) | 34.02  ± 5.90 | 19.03  ± 1.85 | 58.55  ± 3.20 | | 19.77  ± 2.83 | 8.80  ± 0.88 | | 25.01  ± 5.56 | 54.43  ± 2.47 | | | 25.39  ± 3.83 | | 48.19  ± 2.43 | | | 17.97  ± 2.15 | | 53.27  ± 1.63 | | | 20.59  ± 1.72 | | |  | | 20.70  ± 3.13 | |  |
| CIV  (I) | 42.96  ± 8.07 | 28.95  ± 2.38 | 69.23  ± 3.48 | | 24.99  ± 2.93 | 11.59  ± 1.28 | | 29.20  ± 3.14 | 59.33  ± 1.97 | | | 29.97  ± 2.13 | | 58.89  ± 2.22 | | | 31.68  ± 4.02 | | 59.71  ± 1.49 | | | 24.43  ± 2.70 | | | 72.63  ± 5.64 | | 19.19  ± 2.27 | |  |
| CIV  (D) | 51.31  ± 9.03 | 28.64  ± 2.18 | 56.30  ± 3.03 | | 31.54  ± 4.23 | 11.92  ± 1.56 | | 26.75  ± 5.74 | 61.48  ± 1.38 | | | 24.47  ± 1.91 | | 60.69  ± 1.70 | | | 25.98  ± 3.55 | | 57.59  ± 2.28 | | | 33.57  ± 3.65 | | |  | | 25.29  ± 2.27 | |  |
| ATPsyn  (S) | 7.31  ± 1.47 | 11.32  ± 3.21 | 9.90  ± 2.85 | | 11.93  ± 4.33 | 11.75  ± 3.16 | | 8.28  ± 2.19 | 7.82  ± 1.64 | | | 16.74  ± 4.78 | | 11.46  ± 3.31 | | | 18.28  ± 2.98 | | 5.90  ± 1.06 | | | 22.99  ± 2.76 | | |  | | 23.53  ± 1.76 | |  |
| ATPsyn  (I) | 6.88  ± 1.57 | 10.05  ± 2.07 | 4.43  ± 1.06 | | 10.67  ± 2.81 | 11.87  ± 2.08 | | 8.32  ± 3.55 | 7.69  ± 1.34 | | | 13.94  ± 3.66 | | 6.83  ± 1.64 | | | 19.21  ± 5.60 | | 6.30  ± 1.36 | | | 18.07  ± 2.67 | | | 6.10  ± 2.50 | | 20.02  ± 2.19 | |  |
| ATPsyn  (D) | 10.74  ± 3.61 | 6.96  ± 2.64 | 9.61  ± 2.53 | | 10.42  ± 3.17 | 6.86  ± 1.42 | | 5.34  ± 1.36 | 5.29  ± 0.91 | | | 11.34  ± 3.65 | | 5.28  ± 1.06 | | | 11.28  ± 2.02 | | 7.77  ± 1.99 | | | 14.28  ± 3.20 | | |  | | 12.04  ± 2.15 | |  |
| ***Fatty acid metabolism*** | | | | | | | | | | |  | |  | | |  | |  | | |  | |  | | |  | |  |  |
| HOAD  (S) | 15.99  ± 1.14 | 18.76  ± 1.87 | 13.73  ± 1.13 | | 21.41  ± 1.78 | 50.52  ± 1.75 | | 28.36  ± 2.55 | 10.29  ± 0.81 | | | 15.33  ± 2.09 | | 12.84  ± 0.85 | | | 17.25  ± 1.15 | | 12.40  ± 0.78 | | | 15.53  ± 2.01 | | |  | | 13.07  ± 1.15 | |  |
| HOAD  (I) | 16.61  ± 1.96 | 25.44  ± 1.73 | 9.39  ± 1.51 | | 29.69  ± 0.95 | 50.41  ± 1.02 | | 28.58  ± 3.96 | 7.10  ± 0.51 | | | 17.96  ± 2.67 | | 11.20  ± 1.07 | | | 21.41  ± 1.04 | | 9.58  ± 0.64 | | | 17.96  ± 2.67 | | | 11.29  ± 0.53 | | 16.75  ± 1.30 | |  |
| HOAD  (D) | 10.20  ± 2.52 | 23.43  ± 1.27 | 11.47  ± 0.53 | | 23.02  ± 1.23 | 57.69  ± 0.80 | | 31.01  ± 1.80 | 8.12  ± 0.59 | | | 15.56  ± 0.86 | | 11.18  ± 0.77 | | | 19.37  ± 1.64 | | 13.25  ± 1.85 | | | 15.75  ± 0.97 | | |  | | 11.37  ± 2.24 | |  |
| ***Adenylate metabolism*** | | | | | | | | | | |  | |  | | |  | |  | | |  | |  | | |  | |  |  |
| AK  (S) | 228.52  ± 5.89 | 200.90  ± 6.49 | 221.54  ± 10.98 | | 265.82  ± 11.26 | 206.00  ± 6.37 | | 196.98  ± 12.89 | 304.16  ± 7.86 | | | 237.02  ± 7.05 | | 291.20  ± 7.19 | | | 304.65  ± 5.60 | | 273.93  ± 21.32 | | | 235.30  ± 4.90 | | |  | | 225.19  ± 5.66 | |  |
| AK  (I) | 211.383  ± 6.73 | 178.12  ± 7.45 | 228.93  ± 13.88 | | 280.38  ± 8.08 | 192.81  ± 10.97 | | 157.43  ± 9.52 | 258.16  ± 7.98 | | | 236.21  ± 8.43 | | 270.90  ± 9.81 | | | 290.34  ± 8.42 | | 257.36  ± 25.19 | | | 221.85  ± 5.58 | | | 296.86  ± 47.21 | | 217.51  ± 4.72 | |  |
| AK  (D) | 214.85  ± 23.14 | 169.73  ± 7.22 | 286.48  ± 18.84 | | 249.91  ± 12.93 | 142.77  ± 6.96 | | 166.79  ± 6.93 | 240.85  ± 8.28 | | | 249.33  ± 5.53 | | 245.21  ± 15.92 | | | 201.36  ± 11.85 | | 221.94  ± 14.64 | | | 246.04  ± 5.52 | | |  | | 254.59  ± 4.71 | |  |
| CK  (S) | 68.80  ± 2.49 | 33.46  ± 1.16 | 70.16  ± 7.37 | | 42.15  ± 3.55 | 63.51  ± 4.71 | | 35.01  ± 3.54 | 69.46  ± 5.99 | | | 36.16  ± 5.11 | | 64.57  ± 11.68 | | | 38.94  ± 4.76 | | 83.71  ± 9.61 | | | 49.47  ± 5.68 | | |  | | 12.39  ± 1.30 | |  |
| CK  (I) | 59.74  ± 6.83 | 32.67  ± 1.70 | 71.87  ± 5.38 | | 42.55  ± 6.37 | 61.75  ± 3.26 | | 20.49  ± 1.93 | 67.31  ± 1.36 | | | 22.03  ± 4.63 | | 61.06  ± 3.03 | | | 32.17  ± 2.54 | | 84.58  ± 5.75 | | | 40.58  ± 7.17 | | | 26.79  ± 1.17 | | 16.42  ± 1.63 | |  |
| CK  (D) | 68.23  ± 8.09 | 43.85  ± 2.21 | 72.32  ± 5.04 | | 44.17  ± 8.55 | 31.87  ± 5.26 | | 35.37  ± 9.76 | 64.27  ± 3.95 | | | 25.95  ± 4.75 | | 65.97  ± 7.54 | | | 26.19  ± 5.45 | | 62.93  ± 4.91 | | | 35.64  ± 4.48 | | |  | | 12.50  ± 3.06 | |  |

Values are given in as the mean ± SEM. List of abbreviations: HA = high altitude; LA = low altitude; S = tissue sampled from surface muscle; I = tissue sampled from intermediate muscle; D = tissue sampled from deep muscle; Mb = myoglobin; HK = hexokinase; PK = pyruvate kinase; LDH = lactate dehydrogenase; CS = citrate synthase; IDH = isocitrate dehydrogenase; MDH = malate dehydrogenase; CI = complex 1 (syn. NADH:ubiquinone oxidoreductase); CII = complex 2 (syn. succinate dehydrogenase); CIV = complex IV (syn. cytochrome c oxidase); ATPsyn = F_O_F_1_ ATP synthase; HOAD = 3-hydroxyacyl-CoA dehydrogenase; AK = adenylate kinase; and CK = creatine kinase.
