# Supplementary material for: Convergent changes in muscle metabolism depend on duration of high-altitude ancestry across Andean waterfowl
Source: eLife. 2020 Jul 30;9:e56259. doi: 10.7554/eLife.56259 (PMC7494360; doi:10.7554/eLife.56259)
Supplement: Transparent reporting form [file elife-56259-transrepform.pdf]

eLIFE
1st Floor
24 Hills Road
Cambridge CB2 1JP, UK
P 01223 855340
W elifesciences.org
T @elife

eLife's transparent reporting form

We encourage authors to provide detailed information *within their submission* to facilitate the interpretation and replication of experiments. Authors can upload supporting documentation to indicate the use of appropriate reporting guidelines for health-related research (see EQUATOR Network), life science research (see the BioSharing Information Resource), or the ARRIVE guidelines for reporting work involving animal research. Where applicable, authors should refer to any relevant reporting standards documents in this form.

If you have any questions, please consult our Journal Policies and/or contact us: editorial@elifesciences.org.

Sample-size estimation

- You should state whether an appropriate sample size was computed when the study was being designed
- You should state the statistical method of sample size computation and any required assumptions
- If no explicit power analysis was used, you should describe how you decided what sample (replicate) size (number) to use

Please outline where this information can be found within the submission (e.g., sections or figure legends), or explain why this information doesn't apply to your submission:

The number of animals used in this experiment was determined by the number of animals we were allowed to sample as stipulated in the animal collection and tissue export permits we received. For more detailed information about the permits obtained to conduct this research please refer to the main text in the 'Materials and Methods' section under 'Tissue sampling'. Our experiment uses similar sample sizes as described in Natarajan et al., 2015 (https://doi.org/10.1371/journal.pgen.1005681). More detailed information on sample-sizes can be found in the 'Materials and Methods' section under 'Tissue sampling'.

Replicates

- You should report how often each experiment was performed
- You should include a definition of biological versus technical replication
- The data obtained should be provided and sufficient information should be provided to indicate the number of independent biological and/or technical replicates
- If you encountered any outliers, you should describe how these were handled
- Criteria for exclusion/inclusion of data should be clearly stated
- High-throughput sequence data should be uploaded before submission, with a private link for reviewers provided (these are available from both GEO and ArrayExpress)

Please outline where this information can be found within the submission (e.g., sections or figure legends), or explain why this information doesn't apply to your submission:

eLife Sciences Publications, Ltd is a limited liability non-profit non-stock corporation incorporated in the State of Delaware, USA, with company number 5030732, and is registered in the UK with company number FC030576 and branch number BR015634 at the address 1st Floor, 24 Hills Road, Cambridge CB2 1JP | August 2014

In all cases, we were limited to taking a single sample from each individual animals sampled by our permits. For enzyme analysis, homogenetes from each animal were plated in triplicate as technical replicates. The number of biological replicates is given by the N values in the paper. This is the standard number of replicates used for the experiments conducted in this study (see above answer for an example reference from the literature). We did not remove any 'outliers' from the data. This information can be found in the 'Materials and Methods' section under 'Enzyme activities and myoglobin assays'. All new nucleotide sequences used in this experiment were deposited in GenBank and the accession numbers are provided in the supplementary materials (Table S9).

eLife Sciences Publications, Ltd is a limited liability non-profit non-stock corporation incorporated in the State of Delaware, USA, with company number 5030732, and is registered in the UK with company number FC030576 and branch number BR015634 at the address 1st Floor, 24 Hills Road, Cambridge CB2 1JP | August 2014

Statistical reporting

- Statistical analysis methods should be described and justified
- Raw data should be presented in figures whenever informative to do so (typically when N per group is less than 10)
- For each experiment, you should identify the statistical tests used, exact values of N, definitions of center, methods of multiple test correction, and dispersion and precision measures (e.g., mean, median, SD, SEM, confidence intervals; and, for the major substantive results, a measure of effect size (e.g., Pearson's r, Cohen's d)
- Report exact p-values wherever possible alongside the summary statistics and 95% confidence intervals. These should be reported for all key questions and not only when the p-value is less than 0.05.

Please outline where this information can be found within the submission (e.g., sections or figure legends), or explain why this information doesn't apply to your submission:

Detailed information regarding sample size (N), capture range, and estimates of time since divergence for each species tested in this study can be found in Table 1. Detailed information about the statistical results is reported in the supplementary materials in tables S1-S7. This includes p-values, exact values of N, R^2, correlation coefficients, and raw data values.

(For large datasets, or papers with a very large number of statistical tests, you may upload a single table file with tests, Ns, etc., with reference to sections in the manuscript.)

Group allocation

- Indicate how samples were allocated into experimental groups (in the case of clinical studies, please specify allocation to treatment method); if randomization was used, please also state if restricted randomization was applied
- Indicate if masking was used during group allocation, data collection and/or data analysis

Please outline where this information can be found within the submission (e.g., sections or figure legends), or explain why this information doesn't apply to your submission:

Information on how samples were allocated into groups is reported in Table 1 and can be found in greater detail in the 'Materials and Methods' section under 'tissue sampling'.

Additional data files ("source data")

- We encourage you to upload relevant additional data files, such as numerical data that are represented as a graph in a figure, or as a summary table
- Where provided, these should be in the most useful format, and they can be uploaded as "Source data" files linked to a main figure or table
- Include model definition files including the full list of parameters used
- Include code used for data analysis (e.g., R, MatLab)
- Avoid stating that data files are "available upon request"

Please indicate the figures or tables for which source data files have been provided:

The data used to create all figures for this study is included in the supplementary files in tables S1-S7. Included in the supplementary files is also a full list of all statistical results and parameters including F, N, p-values, and degrees of freedom used. Detailed descriptions of the parameters used for each analysis is described in the relevant sub section under 'materials and methods' and more detailed descriptions can be found in the supplementary materials.
